# The Impact of Red Yeast Rice Extract Use on the Occurrence of Muscle Symptoms and Liver Dysfunction: An Update from the Adverse Event Reporting Systems and Available Meta-Analyses

**DOI:** 10.3390/nu16030444

**Published:** 2024-02-02

**Authors:** Giuseppe Danilo Norata, Maciej Banach

**Affiliations:** 1 Department of Pharmacological and Biomolecular Sciences, University of Milan, 20133 Milan, Italy; danilo.norata@unimi.it; 2 Center for the Study of Atherosclerosis, Bassini Hospital, 20092 Cinisello Balsamo, Italy; 3 Department of Preventive Cardiology and Lipidology, Medical University of Lodz (MUL), 90-419 Lodz, Poland

**Keywords:** monacolin K, red yeast rice, lipid lowering, LDL-C, muscle adverse events, liver adverse events

## Abstract

Red yeast rice (RYR) has a cholesterol-lowering effect due to the presence of bioactive components (monacolins, mainly monacolin K) that act by inhibiting the activity of 3-hydroxy-3-methylglutaryl coenzyme A (HMG-CoA) reductase. The European Food Safety Authority (EFSA) assessed the use of RYR and, while pointing out several uncertainties regarding the available data, raised a warning related to the safety of RYR when used as a food supplement at a dose of monacolin as low as 3 mg/day. In their decision in June 2023, EFSA approved the use of monacolins from RYR at doses less than 3 mg/day. We therefore decided to interrogate the different adverse event reporting systems (FAERS and CAERS) and analyse the characteristics of the cases reported to be associated with RYR supplements, and we reviewed the most recent meta-analyses with a focus on the occurrence of muscle symptoms and liver dysfunction. In terms of all musculoskeletal disorders from September 2013 (when the first case related to RYR consumption was recorded) to 30 September 2023, 363,879 cases were reported in the FAERS, with the number of cases related to RYR consumption being very small and accounting for 0.008% of cases. In the same time frame, 27,032 cases of hepatobiliary disorders were reported, and the cases attributable to RYR ingestion accounted for 0.01% of all cases. A low rate of muscle symptoms and liver dysfunction attributed to RYR ingestion was also observed in the CAERS database, where only 34 cases of adverse muscle events and 10 cases of adverse liver events reported RYR as the suspect product, while 19 cases of both muscle events and 10 cases of adverse liver events reported it as a concomitant product. This profile mirrors that of meta-analyses of randomised clinical trials of RYR, in which RYR use was not associated with either liver dysfunction or muscular adverse symptoms.

## 1. Introduction

Red yeast rice (RYR) is a traditional Chinese product obtained by fermenting rice with *Monascus purpureus*, a type of red yeast [1]. RYR has been used for centuries in traditional Chinese medicine and is also used as a dietary supplement [2].

Several compounds are produced during the fermentation process [3]. RYR contains naturally occurring compounds known as monacolins, which are chemically identical to statins, a class of prescription drugs used to lower cholesterol levels. One of the main components of red yeast rice is monacolin K, which is chemically identical to lovastatin, a prescription drug used to lower circulating cholesterol levels [1]. For this reason, RYR is often promoted as a natural remedy for high cholesterol. Some studies have indeed shown that RYR can lower LDL-C levels. A meta-analysis showed that RYR supplements significantly reduced total cholesterol, LDL-C, and triglycerides while increasing HDL-C [4].

However, the use of RYR as a cholesterol-lowering dietary supplement has raised concerns and regulatory issues, primarily because the monacolin K content can vary significantly between different products. In the United States, the Food and Drug Administration (FDA) has raised concerns about the safety and consistency of red yeast rice supplements and has issued warnings against its use, despite the fact that the FDA decision has not been updated for the last 16 years [5].

The European Food Safety Authority (EFSA) has evaluated the safety and efficacy of RYR supplements for managing cholesterol levels. In 2018, EFSA published a scientific opinion on the use of monacolin K from red yeast rice in food supplements [6]. Monacolin K is the active ingredient in RYR and is chemically identical to lovastatin [6]. The use of RYR as a dietary supplement may therefore lead to people being exposed to monacolin K at levels within the recommended therapeutic dose range of lovastatin, which is from 10 to 80 mg/day [6]. Therefore, consumption of RYR can potentially lead to muscle-related symptoms similar to the muscle-related side effects of statin-based therapies [7], which can range from mild muscle discomfort to more severe muscle pain, known as myopathy. In addition, monacolin K can have similar effects on the liver as statins [8], and in some cases, it has caused liver damage [9], despite the fact that a causal relation between any liver harm and statin therapy has never been confirmed. The occurrence of myalgia or muscle pain with RYR varies from person to person as it does with statin drugs. Some people experience no muscle-related side effects, while others are more prone to these issues, but altogether, the statin-related muscle symptoms (SAMS) prevalence is only about 9%, between 5.9 and 7% when diagnosed with approved definitions, and only about 1–3% for complete statin intolerance [10]. This makes these drugs some of the best-tolerated in cardiology. There may be a genetic component that influences a person’s susceptibility to these side effects. It is worth noting that RYR can interact with other drugs, including other lipid-lowering therapies, which can be potentially harmful [11]. Furthermore, the monacolin content in RYR supplements can vary from brand to brand and even from batch to batch; this variability can make it challenging to predict the effects on cholesterol levels and potential side effects [12]. Thus, it is a strong recommendation to consider only the products from trusted manufacturers with a confirmed high-quality production process.

Based on the available information on possible adverse effects observed in individuals taking the RYR supplement, it appeared that monacolin K at a dose of 10 mg/day may raise safety concerns; in addition, serious adverse events have been reported by some authors even at a dose of 3 mg/day [6]. For this reason, as mentioned above, the European Commission declared in 2022 that RYR products must contain less than 3 mg of monacolins for daily consumption [13].

In a previous study, we analysed adverse event reporting systems and available case reports and concluded that the occurrence of rhabdomyolysis or severe acute hepatitis that could be associated with the use of RYR appears to be extremely rare compared to the occurrence with statins (which is rare to common) [14].

In this paper, we discuss the available data from adverse event reporting systems and meta-analyses on the use of RYR on the occurrence of muscle symptoms and liver dysfunction.

## 2. Sources of Data

The FDA Adverse Event Reporting System (FAERS) Public Dashboard and the CFSAN Adverse Event Reporting System (CAERS) database are described in a previous paper [14]. In brief, FAERS (https://open.fda.gov/data/faers/, accessed on 3 November 2023) is a web-based tool that enables researchers to obtain information on adverse events reported to the FDA by the pharmaceutical industry, healthcare providers, and consumers, while CAERS (https://open.fda.gov/data/caers/, accessed on 3 November 2023) is a database containing information on adverse events and product complaints submitted to the FDA regarding foods, dietary supplements, and cosmetics. Adverse events and medication errors are coded using terms in the Medical Dictionary for Regulatory Activities (MedDRA) terminology.

Both adverse event reporting systems (FAERS and CAERS) were queried to investigate the adverse effects associated with the use of RYR use, focusing on adverse events related to muscles and liver.

Liver injury is classified as hepatic damage, liver damage aggravated, metabolic liver injury, injury to the liver, acute liver injury, liver damage, hepatic damage, chronic liver injury, acute liver damage, and damaged liver. Muscle disorders are defined as a subclass of musculoskeletal and connective tissue disorders and include myopathies, muscle-related signs and symptoms NEC (not elsewhere classified), muscle pains, and muscle weakness.

## 3. Analysis of FAERS Database

**Figure 1:**
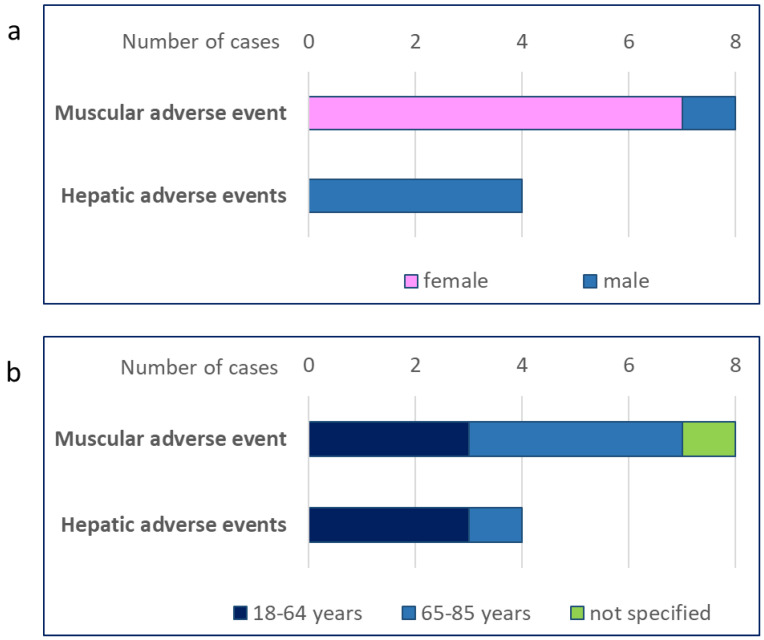
Muscular and hepatic adverse events from the FAERS database. The number of muscular or hepatic adverse events in people taking red yeast rice was retrieved from the FDA Adverse Event Reporting System (FAERS) database and analysed by gender (**a**) and age (**b**).

As of 30 September 2023, the number of adverse events received by the FDA for drugs and therapeutic biological products was 27,634,809 (20,310,452 from 2013 onward). Of these, 28 cases of adverse events (25 of which were classified as serious events) in people taking RYR were reported in the FAERS database (Supplementary Table S1). These included eight cases of musculoskeletal and connective tissue disorders, including myopathy (five cases) and rhabdomyolysis (four cases) [14] (Table 1, Supplementary Table S2). These events occurred in seven women and one man (Figure 1a); the age distribution is shown in Figure 1b. Musculoskeletal disorders associated with RYR use accounted for 0.002% of all cases of musculoskeletal disorder reported in the FAERS database up to 30 September 2023 (402,758).

Four cases of hepatobiliary disorders associated with RYR use were also reported. More specifically, hepatic cytolysis was reported in three cases and liver injury in one case (Table 1). Of note, we also found one case of transaminase elevation in a person with hepatitis B taking RYR. All of these adverse events occurred in men, four of whom were aged <65 years old (Figure 1a,b). Supplementary Table S2 shows the distribution of the individual adverse events by gender and age. The analysis of data from FAERS revealed a total of 28,133 cases of hepatobiliary disorders, as defined for this analysis, from 2013 onward (the date of the first report for RYR); thus, cases of liver disorders attributable to RYR use accounted for 0.014%.

A detailed description of cases of adverse events in people taking RYR can be found in Supplementary Tables S3 and S4.

## 4. Analysis of CAERS Database

**Figure 2:**
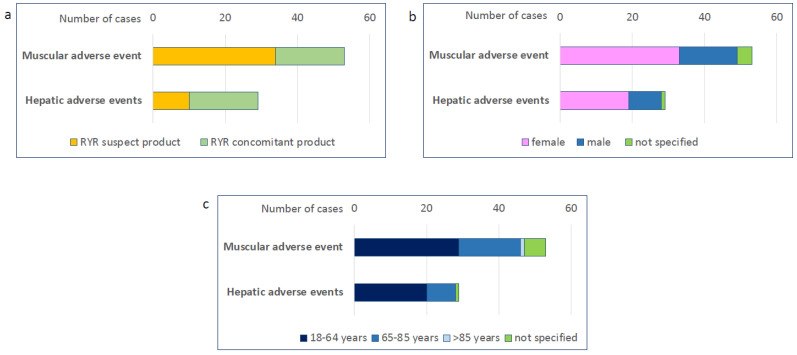
Muscular and hepatic adverse events from the CAERS database. The number of muscular or hepatic adverse events in people taking red yeast rice was retrieved from the CFSAN Adverse Event Reporting System (CAERS) database and analysed by suspect product (**a**), gender (**b**), and age (**c**).

As of June 2023, a total of 1,301,786 cases of adverse events were reported in the CAERS database. We identified a total of 223 cases of adverse events in people taking RYR. These included 53 cases of muscle-related adverse events and 29 cases of liver-related adverse events. In 34 cases of muscle-related adverse events and 10 cases of liver-related adverse events, RYR was reported as the suspected product, while in all other cases it was reported as a concomitant product (Figure 2a). Most adverse events occurred in women (Figure 2b) and in people aged 18–64 years (Figure 2c).

Muscle adverse events identified in the CAERS database included muscle disorders, muscle fatigue, muscle spasms, muscle tightness, muscular weakness, musculoskeletal chest pain, musculoskeletal discomfort, musculoskeletal pain, musculoskeletal stiffness, myalgia, myopathy, pain in an extremity, and rhabdomyolysis. The most common adverse events were myalgia and muscle spasms (Table 2). Hepatic adverse events were reported as an increase in hepatic enzymes or abnormal liver function test results, hepatic failure, hepatic pain, hepatomegaly, and liver injury, with abnormal liver function test results being the most common (Table 3). It is worth noting that different adverse reactions, either muscular or hepatic, often occurred in the same person (Supplemental Tables S5 and S6).

However, we must emphasise that a number of these reports were related to the same product in the same time period (Supplementary Tables S5 and S6). Both muscular and hepatic adverse events were reported in people taking a vitamin B supplement, which was considered the suspect product, rather than RYR, which was labelled as the concomitant product. Following several reports of adverse events associated with the use of this product (including fatigue, muscle cramping, and myalgia, as well as abnormal laboratory findings for liver and thyroid function), a preliminary FDA laboratory analysis revealed the presence of two potentially harmful ingredients that were not listed on the label and that should not be included in a dietary supplement (https://www.safemedicines.org/2013/07/fda-alert-healthy-life-chemistry-by-purity-first-b-50-fda-health-risk-warning-undeclared-ingredients.html, accessed on 3 November 2023).

Supplementary Tables S5 and S6 contain a detailed description of all cases.

## 5. Results from Meta-Analyses

An analysis of 13 randomised clinical trials with 804 participants showed that RYR was effective in reducing total cholesterol, LDL-C, and triglycerides, but no serious adverse effects were recorded [15]. It is noteworthy that the dose of monacolin K in the studies included in this meta-analysis ranged from 2 mg to 11.4 mg (some lacked this information). Serum alanine transaminase and aspartate aminotransferase levels were significantly elevated in the intervention group but were within the normal range; RYR did not increase creatine kinase levels or blood glucose compared to placebo [15]. A subsequent meta-analysis of 20 RCTs involving 6663 patients (with a range of monacolin K from 2.4 mg up to 24 mg) reported that the incidence of kidney injury and liver abnormalities was less than 5% in both the RYR and placebo groups; the incidence of developing muscle symptoms ranged from 0 to 23.8% in the RYR group and from 0% to 36% in the placebo group [16]. No rhabdomyolysis or myopathy with elevated creatine kinase >10 times the upper limit of normal was observed in any study [16]. In a study comparing myopathies in the RYR group with pravastatin, the risk of developing muscle symptoms was lower in the RYR group [16].

Of note, another meta-analysis of studies in which treatment with RYR supplements (containing varying amounts of monacolin K—from 2 to 10 mg—along with additional ingredients) was administered over a period of 4 to 24 weeks showed no changes in liver or kidney function in the RYR or control group [17]; also, no significant changes in creatine kinase levels or distressing symptoms were reported with RYR supplementation [17].

A meta-analysis of 53 randomised clinical trials involving 8535 subjects was conducted to assess the safety of RYR supplementation in short- and longer-term studies [18]. There was no evidence of an increased risk of RYR-associated musculoskeletal disorders (even when analysed by monacolin K daily dose of ≤3, 3.1–5, or >5 mg/day), and no non-musculoskeletal adverse events (including adverse cardiovascular events, gastrointestinal disorders, urinary tract infections, general discomfort, arthralgia, or mildly elevated hepatic or renal biomarkers) or serious adverse events that required urgent medical interventions and/or hospitalisation, were life-threatening, or resulted in death were observed [18].

Mazzanti et al. reported hepatic adverse events in 10 people who had taken a red yeast rice supplement [19]. Four of them experienced an increase in liver enzyme levels and six had acute hepatitis requiring hospitalisation. The limitation of this small case study was that the authors did not confirm the causality between RYR and the observed adverse events, and they did not adjust any other risk factors or conditions that might have been responsible for this [19]. A post-marketing analysis of dietary supplements containing red yeast rice found that hepatic adverse events (including transaminase elevations) occurred in 26 of 855 reported adverse events (3%), with a frequency of 0.0011% among those exposed to red yeast rice.

To date, the results of all available meta-analyses indicate a neutral effect of RYR supplementation on parameters associated with liver or muscle dysfunction, an interesting observation considering that most studies were conducted with a dose of monacolin K greater than 3 mg.

## 6. Limitations

It is crucial that we recognise some limitations. Firstly, the adverse event reporting systems on which we rely operate on a voluntary basis and accept reports from consumers, healthcare professionals, and manufacturers, along with mandatory reports from manufacturers of dietary supplements. It is important to note that the mere presence of reports of a particular drug or biologic in these systems does not automatically establish a causal link between the product and the adverse event. The FDA does not require that a causal relationship be established, and the reports may not be detailed enough to make a comprehensive assessment.

It is also important to understand that the FDA does not receive reports for every adverse event or medication error associated with a product. Duplicate reports may occur, especially if both a consumer and a manufacturer submit the same report. Some reports may lack necessary information, and the content is not verified, leading to uncertainty as to the actual cause of the adverse event.

Also, when looking at adverse event reports in the CAERS database, the total number of reports on a product does not represent a definitive conclusion by the FDA regarding the product’s role in causing adverse events. It is important to understand that while these reports provide information, they do not establish causation. Reported events could be related to underlying medical conditions, activities, or concomitant use of other products, or could have occurred by chance.

Reports submitted to the FDA vary in the quality and reliability of the information provided. Some reports may lack relevant data, such as details about the individual’s concurrent medical conditions or concomitant use of other products or medications. Importantly, the information in these reports has not been scientifically tested for causality and cannot be used to estimate the actual incidence or risk associated with a particular product. In this context, we must recognise the lack of valuable tools for the assessment of causality.

## 7. Conclusions

In this paper, we reviewed the available data from the adverse event reporting systems (FAERS and CAERS) and published meta-analyses on the association between the use of RYR extracts and the occurrence of musculoskeletal symptoms and liver dysfunction. In terms of all musculoskeletal disorders from September 2013 (when the first case related to RYR consumption was recorded) to 30 September 2023, 402,758 cases were reported in the FAERS, with the cases related to RYR consumption being very rare and accounting for 0.007% of cases. In the same time frame, 28,133 cases of hepatobiliary disorders were reported, and cases attributable to RYR ingestion accounted for 0.01% of all cases. A low rate of muscle symptoms and liver dysfunction attributed to RYR ingestion was also observed in the CAERS database, where only 34 cases of adverse muscle events and 10 cases of adverse liver events reported RYR as the suspect product, while 19 cases of both muscle events and 10 cases of adverse liver events reported it as a concomitant product. It is worth noting that the FAERS and CAERS databases do not allow evaluation of the daily amount of RYR associated with the adverse events. However, from the names of the RYR-containing products entered into the CAERS system, one could infer that most cases are well above the recommended dose of monacolin of 3 mg/day. This would also suggest that the number of cases attributable to RYR products with a monacolin limit within the current EFSA regulation will definitely be lower. In addition, for some RYR-containing products in the CAERS database, it was later determined by the FDA that other impurities may have been responsible for the reported adverse events (https://www.safemedicines.org/2013/07/fda-alert-healthy-life-chemistry-by-purity-first-b-50-fda-health-risk-warning-undeclared-ingredients.html, accessed on 3 November 2023), although these cases are still included in the CAERS database.

Again, this limits the number of “true” cases attributable to RYR use. This profile mirrors that of meta-analyses of randomised clinical trials of RYR, in which RYR use was not associated with either liver dysfunction or muscular adverse symptoms. These results are all the more interesting as most of the clinical trials were conducted with a dose of more than 3 mg monacolin K.

Overall, the data from randomised clinical trials and adverse event reporting systems minimise the risk of muscular and hepatic adverse events associated with the use of RYR—a risk that is even lower considering the current indications from EFSA of 3 mg of monacolin K per day.

## Figures and Tables

**Table 1:** Number of cases of musculoskeletal and connective tissue disorders and hepatobiliary disorders in people taking red yeast rice (FAERS database). Note that a case may have one or more reported outcomes.

	In People Taking RYR	Total in FAERS	% of RYR-Associated Adverse Events
**Musculoskeletal and connective tissue disorders**	**8**	**402,758**	**0.002%**
Myopathy	5	4823	0.1%
Rhabdomyolysis	4	21,605	0.019%
Pain in extremity	1	199,374	0.0005%
Muscular weakness	1	71,270	0.0014%
Myalgia	1	104,736	0.001%
Sacral pain	1	950	0.105%
**Hepatobiliary disorders**	**4**	**28,133**	**0.014%**
Hepatic cytolysis	3	15,215	0.02%
Liver injury	1	12,918	0.008%

**Table 2:** Number of cases of muscular adverse events in people taking red yeast rice versus total cases in the CAERS database.

Adverse Event	In People Taking RYR	Total in CAERS	% of RYR-Associated Adverse Events
**Musculoskeletal disorders**	**53**		
Muscle disorders	3	24	12.5%
Muscle fatigue	1	34	2.94%
Muscle spasms	29	1107	2.62%
Muscle tightness	1	45	2.22%
Muscular weakness	6	295	2.03%
Musculoskeletal chest pain	1	48	2.08%
Musculoskeletal discomfort	1	22	4.55%
Musculoskeletal pain	1	104	0.96%
Musculoskeletal stiffness	2	181	1.10%
Myalgia	29	753	3.85%
Myopathy	2	5	40.0%
Pain in extremity	3	395	0.76%
Rhabdomyolysis	3	242	1.24%

**Table 3:** Number of cases of hepatic adverse events in people taking red yeast rice versus total cases in the CAERS database.

Adverse Event	In People Taking RYR	Total in CAERS	% of RYR-Associated Adverse Events
Alanine aminotransferase increased	3	474	0.63%
Aspartate aminotransferase increased	2	459	0.44%
Hepatic enzyme increased	4	716	0.56%
Hepatic failure	1	231	0.43%
Hepatic pain	1	57	1.75%
Hepatomegaly	1	91	1.10%
Liver function test abnormal	21	536	3.92%
Liver injury	1	398	0.25%

## Data Availability

Data are contained within the article and supplementary materials.

## Supplementary Materials

The following supporting information can be downloaded at: https://www.mdpi.com/article/10.3390/nu16030444/s1, Table S1: Number of adverse event cases in people taking red yeast rice from the FAERS database; Table S2: Number of muscular and hepatic adverse event cases in people taking red yeast rice from the FAERS database analysed by gender and age; Table S3: Details of muscular adverse event reports in people taking red yeast rice (FAERS database); Table S4: Details of hepatic adverse event reports in people taking red yeast rice (FAERS database); Table S5: Details of muscular adverse event reports in people taking red yeast rice (CAERS database); Table S6: Details of hepatic adverse event reports in people taking red yeast rice (CAERS database).
