# Supplementary material for: The Impact of Red Yeast Rice Extract Use on the Occurrence of Muscle Symptoms and Liver Dysfunction: An Update from the Adverse Event Reporting Systems and Available Meta-Analyses
Source: Nutrients. 2024 Feb 2;16(3):444. doi: 10.3390/nu16030444 (PMC10857633; doi:10.3390/nu16030444)
Supplement: Supplementary file 1 [file nutrients-16-00444-s001.zip › nutrients-2811653-supplementary.pdf]

Supplementary Table S1. Number of adverse event cases in people taking red yeast rice from the FAERS database

	**Number of cases**
**Total cases**	**28**
Nervous system disorders	14
General disorders and administration site conditions	13
Musculoskeletal and connective tissue disorders	8
Investigations	5
Hepatobiliary disorders	4
Gastrointestinal disorders	3
Injury, poisoning and procedural complications	2
Psychiatric disorders	2
Metabolism and nutrition disorders	2
Product issues	2
Skin and subcutaneous tissue disorders	1
Blood and lymphatic system disorders	1
Immune system disorders	1

Supplementary Table S2. Number of muscular and hepatic adverse event cases in people taking red yeast rice from the FAERS database analysed by gender and age

		Reaction groups by sex			Reaction groups by age			
	**Number of cases**	**female**	**male**	**not specified**	**18-64 years**	**65-85 years**	**>85 years**	**not specified**
**Musculoskeletal and connective tissue disorders**	**8**	**7**	**1**		**3**	**4**		**1**
Myopathy	5	4	1		1	4		
Rhabdomyolysis	4	4				4		
Pain in extremity	1	1						1
Muscular weakness	1	1			1			
Myalgia	1	1			1			
Sacral pain	1		1		1			
**Hepatobiliary disorders**	**4**		**4**		**3**	**1**		
Hepatic cytolysis	3		3		3			
Liver injury	1		1			1		

Supplementary Table S3. Details of muscular adverse event reports in people taking red yeast rice (FAERS database)

**Suspect Product Active Ingredients**	**Concomitant product**	**Reactions**	**Serious**	**Outcomes**	**Sex**	**Patient Age**	**Reporter Type**	**Country where event occurred**
**Red Yeast;** Simvastatin; Atorvastatin; Rosuvastatin; Fenofibrate	no	**Rhabdomyolysis; Myopathy**	Serious	Other Outcomes	Female	74 YR	Healthcare Professional	US
Pregabalin; Prednisone; Ezetimibe; **Red Yeast**	yes	Feeling abnormal; inflammation; somnolence; bradyphrenia; weight increased; blood test abnormal; **pain in extremity;** gait inability	Non-Serious	Non-Serious	Female	Not Specified	Consumer	US
**Red Yeast;** Aspirin; Atorvastatin; Rosuvastatin; Lovastatin; Pravastatin Sodium; Simvastatin; Colestipol; Dipyridamole	no	**Rhabdomyolysis; myopathy**	Serious	Other Outcomes	Female	77 YR	Healthcare Professional	US
**Red Yeast;** Lovastatin; Pravastatin; Rosuvastatin; Atorvastatin; Cholestyramine; Simvastatin	no	**Rhabdomyolysis; myopathy**	Serious	Other Outcomes	Female	78 YR	Healthcare Professional	IT
Sertraline Hydrochloride; Dietary Supplement; Rosuvastatin Calcium; **Red Yeast**	no	Food interaction; **myopathy; rhabdomyolysis;** drug interaction	Serious	Other Outcomes	Female	70 YR	Healthcare Professional	IT
Atorvastatin Calcium; **Red Yeast**	yes	Blood **creatine phosphokinase increased**; blood creatine phosphokinase Mb increased; **myalgia;** chest discomfort; fatigue; dizziness	Serious	Other Outcomes; Hospitalized	Female	56 YR	Healthcare Professional	CN
Atorvastatin; Fluvastatin Sodium; Simvastatin; **Red Yeast;** Lovastatin	no	**Myopathy; sacral pain**	Serious	Other Outcomes	Male	51 YR	Healthcare Professional	DE
Atorvastatin Calcium; **Red Yeast**	yes	**Muscular weakness**; nausea; balance disorder	Serious	Hospitalized	Female	60 YR	Healthcare Professional	FR

Supplementary Table S4. Details of hepatic adverse event reports in people taking red yeast rice (FAERS database)

Suspect Product Active Ingredients	Concomitant Product Names	Reactions	Serious	Outcomes	Sex	Latest FDA Received Date	Patient Age	Reporter Type	Country where event occurred
**Red Yeast;** Herbals\Turmeric; Atorvastatin	Ramipril; Kardegic	Drug interaction; **hepatic cytolysis**	Serious	Other Outcomes	Male	14-AUG-2019	56 YR	Healthcare Professional	FR
**Red Yeast**; Atorvastatin Calcium; Curcuma Xanthorrhiza Root Extract	Kardegic; Ramipril	**Hepatic cytolysis;** drug interaction	Serious	Other Outcomes	Male	26-MAR-2019	56 YR	Healthcare Professional	FR
**Red Yeast;** Amiodarone Hydrochloride	Nifedipine; Warfarin; Bisoprolol Fumarate	Drug interaction; **liver injury**	Serious	Other Outcomes	Male	05-JAN-2015	68 YR	Healthcare Professional	CN
**Red Yeast;** Tamsulosin Hydrochloride; Allopurinol	Rabeprazole Sodium	Urticaria; **hepatic cytolysis**	Serious	Hospitalized	Male	29-MAY-2013	45 YR	Healthcare Professional	FR

Supplementary Table S5. Details of muscular adverse event reports in people taking red yeast rice (CAERS database)

Date FDA first received report	Product type	PRODUCT	Patient Age	Sex	Reactions	CASE_OUTCOME
7/6/2020	SUSPECT	**Red yeast rice** plus COQ-10	71	Female	Asthenia, diarrhoea, dizziness, hypotension, loss of consciousness, **muscle spasms,** nausea	Other Serious or Important Medical Event
4/16/2019	SUSPECT	**Red yeast rice** 600 mg	73	Female	**Muscle spasms, myalgia**	Hospitalization, Visited Emergency Room
10/11/2018	SUSPECT	**Red yeast rice**	66	Female	Abdominal discomfort, abdominal pain upper, alanine aminotransferase increased, aspartate aminotransferase increased, chest discomfort, chest pain, dyspepsia, epigastric discomfort, eructation, flatulence, gastrointestinal sounds abnormal, headache, malaise, **muscle spasms, myalgia, pain in extremity**	Other Serious or Important Medical Event
5/24/2018	SUSPECT	**Red yeast rice**	61	Male	Amnesia, anaemia, blood potassium increased, bradykinesia, catatonia, claustrophobia, fall, heart rate increased, mydriasis, platelet count increased, **rhabdomyolysis,** weight increased, white blood cell count increased	Life Threatening, Hospitalization, Other Serious or Important Medical Event, Visited Emergency Room
3/13/2018	SUSPECT	**Red yeast rice**	70	Female	Burning sensation, cardiac disorder, dizziness, fall, gout, hypoaesthesia, **musculoskeletal pain,** neuropathy peripheral, pain, psychomotor hyperactivity, red blood cell count increased, somnolence	Hospitalization, Other Serious or Important Medical Event
12/28/2016	SUSPECT	**Red yeast rice** 600 mg	61	Male	Blood pressure increased, cardiac disorder, chest discomfort, confusional state, **myalgia,** myocardial infarction, troponin increased	Hospitalization, Other Serious or Important Medical Event, Visited Emergency Room
5/4/2016	SUSPECT	**Red yeast rice**	63	Female	Chest pain, chest X-ray abnormal, computerised tomogram abnormal, discomfort, dyspnoea, **muscle spasms,** pleural effusion, pneumonia	Other Serious or Important Medical Event, Visited Emergency Room, Visited a Health Care Provider
3/4/2016	SUSPECT	**Red yeast rice**	46	Male	**Muscle disorders**	Other Outcome
11/25/2015	SUSPECT	**Red yeast rice** 600 mg with policosanol	91	Female	Cardiac arrest, cardiac failure, **muscle spasms,** death	Death, Visited a Health Care Provider
6/30/2015	SUSPECT	**Red yeast rice** 600 mg	75	Female	Abasia, asthenia, blood sodium decreased, fatigue, fluid overload, **muscle spasms, nausea,** vomiting	Hospitalization
6/9/2015	SUSPECT	**Red yeast rice** 600 mg	85	Female	Abdominal pain upper, bach pain, compression fracture, intervertebral disc calcification, **musculoskeletal chest pain,** pain	Other Serious or Important Medical Event, Visited Emergency Room, Visited a Health Care Provider
2/23/2015	SUSPECT	**Red yeast rice** 600 mg	78	Male	Abasia, arrhythmia, bradycardia, **rhabdomyolyisis,** swelling	Hospitalization, Visited Emergency Room
2/18/2015	SUSPECT	**Red yeast rice** plus COQ-10	64	Female	Arthralgia, asthenia, cataract, dysstasia, fatigue, gait disturbance, hepatic enzyme increased, **muscular weakness**, **myopathy,** pain	Disability
12/15/2014	SUSPECT	**Red yeast rice**		Male	**Myalgia**	Other Serious or Important Medical Event, Visited a Health Care Provider
7/3/2014	SUSPECT	**Red yeast rice**	58		Arthralgia, asthenia, fatigue, **myalgia**	Other Serious or Important Medical Event
6/30/2014	SUSPECT	**Red yeast rice**	63	Female	**Muscle spasms**, **myalgia**	Other Outcome
9/5/2013	SUSPECT	**Red yeast rice** 600 mg			Anaphylactic reaction, breast mass, **muscle spasms**, pain, pharyngitis	Hospitalization, Visited a Health Care Provider

3/19/2013	SUSPECT	**Red yeast rice** 600 mg	64	Female	Activities of daily living impaired, arthralgia, blood glucose increased, C-reactive protein increased, gait disturbance, glycosylated haemoglobin increased, incontinence, low density lipoprotein increased, **musculoskeletal stiffness**, pain, polyarthritis, red blood cell sedimentation rate increased	Disability, Visited a Health Care Provider
2/27/2013	SUSPECT	**Red yeast rice** 600 mg			Aortic stenosis, asthenia, atrioventricular block first degree, blood pressure increased, body temperature increased, cardiac murmur, chest pain, dyspnoea, erythema, incontinence, lung hyperinflation, malaise, mitral valve incompetence, **myalgia,** osteoarthritis, pain, palpitations, respiration rate increased, sensation of pressure, sinusitis, swelling, tenderness, tenosynovitis, tricuspid valve incompetence, ultrasound doppler abnormal, upper respiratory tract infection	Hospitalization, Required Intervention, Other Serious or Important Medical Event, Visited Emergency Room
3/5/2012	SUSPECT	**Red yeast rice**	79	Male	Asthenia, body temperature increased, carpal tunnel syndrome, malaise, **muscular weakness**, urinary tract infection	Life Threatening, Hospitalization, Required Intervention, Disability, Other Serious or Important Medical Event
11/16/2011	SUSPECT	**Red yeast rice** 600 mg	70	Female	Abdominal pain upper, asthenia, blood urine present, dysphagia, faeces discoloured, gastric haemorrhage, gastritis, **muscle disorder**	Other Serious or Important Medical Event
10/12/2011	SUSPECT	**Red yeast rice**	47	Female	Fatigue, **myalgia,** swelling	Life Threatening
1/3/2011	SUSPECT	**Red yeast rice** 600 mg	44	Male	Asthenia, **myalgia,** tenderness	Visited a Health Care Provider
12/6/2010	SUSPECT	**Red yeast rice** plus COQ-10	64	Male	Anaemia, atrophy, back pain, blood creatinine increased, blood potassium increased, blood urea increased, fibrosis, glomerulosclerosis,haematocrit decreased, haemoglobin decreased, **hepatomegaly,** **muscle spasms**, nephritis interstitial, red blood cell count decreased, renal impairment, renal tubular atrophy, thrombotic microangiopathy	Required Intervention, Visited a Health Care Provider
10/13/2010	SUSPECT	**Red yeast rice**	77	Male	Dyspepsia, **myalgia,** tenderness	Visited a Health Care Provider
2/26/2009	SUSPECT	**Red yeast rice** 600 mg	56	Female	**Muscle tightness**, **muscular weakness**	Other Outcome
7/30/2008	SUSPECT	**Red yeast rice** plus COQ-10	64	Female	Cough, dysstasia, ear discomfort, inflammation, **muscular weakness**, rash, respiratory tract congestion, sinus disorder	Other Serious or Important Medical Event, Other Serious Outcome
7/30/2008	SUSPECT	**Red yeast rice**	70	Female	Arteriosclerosis, carotid artery occlusion, carotid endarterectomy, drug ineffective, **muscle spasms**, **pain in extremity**	Required Intervention, Other Serious or Important Medical Event, Other Serious Outcome
7/17/2008	SUSPECT	**Red yeast rice** plus COQ-10	69	Male	Abasia, abdominal distension, activities of daily living impaired, anal injury, anal sphincter atony, asthenia, diarrhoea, flatulence, gastrointestinal infection, headache, **muscle spasms**, **myalgia,** **myopathy,** nauseas, oedema peripheral, pain swelling, tenderness, toothache	Other Serious or Important Medical Event, Visited Emergency Room, Visited a Health Care Provider, Other Serious Outcome
8/13/2007	SUSPECT	**Red yeast rice** 600 mg	51	Female	**Muscle fatigue**	Other Serious or Important Medical Event, Visited a Health Care Provider
8/28/2006	SUSPECT	**Red yeast rice 600 mg** plus COQ-10 30 mg	55	Female	Fatigue, headache, hypoaesthesia, **myalgia**	Required Intervention, Visited a Health Care Provider
12/9/2005	SUSPECT	**Red yeast rice**		Female	Asthenia, fatigue, **myalgia**	Visited a Health Care Provider
10/6/2005	SUSPECT	**Red yeast rice**	76	Female	Dyspepsia, fatigue, **muscular weakness,** pain	Required Intervention
9/16/2005	SUSPECT	**Red yeast rice** 600 mg		Female	Confusional state, dizziness, fatigue, influenza-like illness, **muscular weakness,** **rhabdomyolysis,** tremor	Other Outcome

11/24/2021	CONCOMITANT	**Red yeast rice**	58	Female	Diarrhoea, faeces discoloured, **muscle spasms**, nausea	Other Serious or Important Medical Event
1/28/2020	CONCOMITANT	**Red yeast rice**	54	Male	**Musculoskeletal discomfort, musculoskeletal stiffness**	Other Serious or Important Medical Event
3/12/2013	CONCOMITANT	**Red yeast rice**	67	Female	Blood cholesterol increased, fatigue, liver function test abnormal, malaise, **muscle spasms, myalgia,** thyroid function test abnormal	Visited a Health Care Provider
3/12/2013	CONCOMITANT	**Red yeast rice**	69	Female	Blood cholesterol increased, fatigue, liver function test abnormal, malaise, **muscle spasms, myalgia,** thyroid function test abnormal	Visited a Health Care Provider
3/12/2013	CONCOMITANT	**Red yeast rice**	45	Female	Blood cholesterol increased, fatigue, liver function test abnormal, malaise, **muscle spasms, myalgia,** thyroid function test abnormal	Visited a Health Care Provider
3/12/2013	CONCOMITANT	**Red yeast rice**	57	Male	Blood cholesterol increased, fatigue, liver function test abnormal, malaise, **muscle spasms, myalgia,** thyroid function test abnormal	Visited a Health Care Provider
3/12/2013	CONCOMITANT	**Red yeast rice**	62	Female	Blood cholesterol increased, fatigue, liver function test abnormal, malaise, **muscle spasms, myalgia,** thyroid function test abnormal	Visited a Health Care Provider
3/12/2013	CONCOMITANT	**Red yeast rice**	77	Female	Blood cholesterol increased, fatigue, liver function test abnormal, malaise, **muscle spasms, myalgia,** thyroid function test abnormal	Visited a Health Care Provider
3/12/2013	CONCOMITANT	**Red yeast rice**	52	Female	Blood cholesterol increased, fatigue, liver function test abnormal, malaise, **muscle spasms, myalgia,** thyroid function test abnormal	Visited a Health Care Provider
3/12/2013	CONCOMITANT	**Red yeast rice**			Blood cholesterol increased, fatigue, liver function test abnormal, malaise, **muscle spasms, myalgia,** thyroid function test abnormal	Visited a Health Care Provider
3/12/2013	CONCOMITANT	**Red yeast rice**	39	Female	Blood cholesterol increased, fatigue, liver function test abnormal, malaise, **muscle spasms, myalgia,** thyroid function test abnormal	Visited a Health Care Provider
3/12/2013	CONCOMITANT	**Red yeast rice**	43	Male	Blood cholesterol increased, fatigue, liver function test abnormal, malaise, **muscle spasms, myalgia,** thyroid function test abnormal	Visited a Health Care Provider
3/12/2013	CONCOMITANT	**Red yeast rice**	46	Male	Blood cholesterol increased, fatigue, liver function test abnormal, malaise, **muscle spasms, myalgia,** thyroid function test abnormal	Visited a Health Care Provider
3/12/2013	CONCOMITANT	**Red yeast rice**	69	Female	Blood cholesterol increased, fatigue, liver function test abnormal, malaise, **muscle spasms, myalgia,** thyroid function test abnormal	Visited a Health Care Provider
3/12/2013	CONCOMITANT	**Red yeast rice**	59	Male	Blood cholesterol increased, fatigue, liver function test abnormal, malaise, **muscle spasms, myalgia,** thyroid function test abnormal	Visited a Health Care Provider
3/12/2013	CONCOMITANT	**Red yeast rice**	58	Male	Blood cholesterol increased, fatigue, liver function test abnormal, malaise, **muscle spasms, myalgia,** thyroid function test abnormal	Visited a Health Care Provider
3/12/2013	CONCOMITANT	**Red yeast rice**	56	Female	Blood cholesterol increased, fatigue, liver function test abnormal, malaise, **muscle spasms, myalgia,** thyroid function test abnormal	Visited a Health Care Provider
3/12/2013	CONCOMITANT	**Red yeast rice**	55	Female	Blood cholesterol increased, fatigue, liver function test abnormal, malaise, **muscle spasms, myalgia,** thyroid function test abnormal	Visited a Health Care Provider
3/28/2008	CONCOMITANT	**Red yeast rice**	60	Female	Blood selenium increased, blood urine present, body temperature increased, cough, diarrhoea, headache, hypotrichosis, jaundice, **muscle spasms**, nausea, pneumonia, skin discolouration	Visited a Health Care Provider

Supplementary Table S6. Details of hepatic adverse event reports in people taking red yeast rice (CAERS database)

Date FDA first received report	Product type	Product	Patient age	Sex	Reactions	CASE_OUTCOME
7/15/2021	SUSPECT	**Red yeast rice**	46	Male	**Hepatic failure**	Life Threatening, Hospitalization, Disability
3/5/2019	SUSPECT	**Red yeast rice**	60	Female	**Hepatic enzyme increased**, renal disorder	Other Serious or Important Medical Event, Visited a Health Care Provider
10/11/2018	SUSPECT	**Red yeast rice**	66	Female	Abdominal discomfort, abdominal pain upper, **alanine aminotransferase increased, aspartate aminotransferase increased,** chest discomfort, chest pain, dyspepsia, epigastric discomfort, eructation, flatulence, gastrointestinal sounds abnormal, headache, malaise, muscle spasms, myalgia, pain in extremity	Other Serious or Important Medical Event
2/18/2015	SUSPECT	**Red yeast rice** with COQ10	64	Female	Arthralgia, asthenia, cataract, dysstasia, fatigue, gait disturbance, **hepatic enzyme increased**, muscular weakness, miopathy, pain	Disability
7/11/2014	SUSPECT	**Red yeast rice**	53	Male	**Liver function test abnormal**	Other Serious or Important Medical Event
7/25/2013	SUSPECT	**Red yeast rice**	69	Female	Chromaturia, faeces discoloured, hypercholesterolaemia, **jaundice, liver injury**	Other Serious or Important Medical Event, Visited a Health Care Provider
6/22/2012	SUSPECT	**Red yeast rice** 600 mg	36	Male	Abdominal pain upper, abdominal pain upper, **alanine aminotransferase increased, aspartate aminotransferase increased, hepatic enzyme increased, hepatic pain, liver function test abnormal**	Other Serious or Important Medical Event
9/21/2011	SUSPECT	**Red yeast rice**	59	Female	Abdominal pain upper, **liver function test abnormal**	Hospitalization
12/6/2010	SUSPECT	COQ -10 plus **Red yeast rice**	64	Male	Anaemia, atrophy, back pain, blood creatinine increased, blood potassium increased, blood urea increased, fibrosis, glomerulosclerosis, haematocrit decreased, haemoglobin decreased, **hepatomegaly,** muscle spasms, nephritis intertitial, red blood cell count decreased, renal impairment, renal tubular atrophy, thrombotic microangiopathy	Required Intervention, Visited a Health Care Provider
11/4/2008	SUSPECT	**Red yeast rice 600 mg**	60	Female	Influenza-like illness, **liver function test abnormal**, loss of consciousness, nausea, syncope	Hospitalization, Other Serious or Important Medical Event, Visited Emergency Room, Visited a Health Care Provider, Other Serious Outcome
8/13/2018	CONCOMITANT	**Red yeast rice**	67	Female	**Liver function test abnormal**	Hospitalization, Other Serious or Important Medical Event, Visited Emergency Room, Visited a Health Care Provider
11/28/2017	CONCOMITANT	**Red yeast rice**	65	Female	**Alanine aminotransferase increased,** respiratory disorder	Disability, Visited a Health Care Provider
3/12/2013	CONCOMITANT	**Red yeast rice**	67	Female	Blood cholesterol increased, fatigue, **liver function test abnormal,** malaise, muscle spasms, myalgia, thyroid function test abnormal	Visited a Health Care Provider
3/12/2013	CONCOMITANT	**Red yeast rice**	69	Female	Blood cholesterol increased, fatigue, **liver function test abnormal,** malaise, muscle spasms, myalgia, thyroid function test abnormal	Visited a Health Care Provider
3/12/2013	CONCOMITANT	**Red yeast rice**	45	Female	Blood cholesterol increased, fatigue, **liver function test abnormal,** malaise, muscle spasms, myalgia, thyroid function test abnormal	Visited a Health Care Provider
3/12/2013	CONCOMITANT	**Red yeast rice**	57	Male	Blood cholesterol increased, fatigue, **liver function test abnormal,** malaise, muscle spasms, myalgia, thyroid function test abnormal	Visited a Health Care Provider

3/12/2013	CONCOMITANT	**Red yeast rice**	62	Female	Blood cholesterol increased, fatigue, **liver function test abnormal,** malaise, muscle spasms, myalgia, thyroid function test abnormal	Visited a Health Care Provider
3/12/2013	CONCOMITANT	**Red yeast rice**	77	Female	Blood cholesterol increased, fatigue, **liver function test abnormal,** malaise, muscle spasms, myalgia, thyroid function test abnormal	Visited a Health Care Provider
3/12/2013	CONCOMITANT	**Red yeast rice**	52	Female	Blood cholesterol increased, fatigue, **liver function test abnormal,** malaise, muscle spasms, myalgia, thyroid function test abnormal	Visited a Health Care Provider
3/12/2013	CONCOMITANT	**Red yeast rice**			Blood cholesterol increased, fatigue, **liver function test abnormal,** malaise, muscle spasms, myalgia, thyroid function test abnormal	Visited a Health Care Provider
3/12/2013	CONCOMITANT	**Red yeast rice**	39	Female	Blood cholesterol increased, fatigue, **liver function test abnormal,** malaise, muscle spasms, myalgia, thyroid function test abnormal	Visited a Health Care Provider
3/12/2013	CONCOMITANT	**Red yeast rice**	43	Male	Blood cholesterol increased, fatigue, **liver function test abnormal,** malaise, muscle spasms, myalgia, thyroid function test abnormal	Visited a Health Care Provider
3/12/2013	CONCOMITANT	**Red yeast rice**	46	Male	Blood cholesterol increased, fatigue, **liver function test abnormal,** malaise, muscle spasms, myalgia, thyroid function test abnormal	Visited a Health Care Provider
3/12/2013	CONCOMITANT	**Red yeast rice**	69	Female	Blood cholesterol increased, fatigue, **liver function test abnormal,** malaise, muscle spasms, myalgia, thyroid function test abnormal	Visited a Health Care Provider
3/12/2013	CONCOMITANT	**Red yeast rice**	59	Male	Blood cholesterol increased, fatigue, **liver function test abnormal,** malaise, muscle spasms, myalgia, thyroid function test abnormal	Visited a Health Care Provider
3/12/2013	CONCOMITANT	**Red yeast rice**	58	Male	Blood cholesterol increased, fatigue, **liver function test abnormal,** malaise, muscle spasms, myalgia, thyroid function test abnormal	Visited a Health Care Provider
3/12/2013	CONCOMITANT	**Red yeast rice**	56	Female	Blood cholesterol increased, fatigue, **liver function test abnormal,** malaise, muscle spasms, myalgia, thyroid function test abnormal	Visited a Health Care Provider
3/12/2013	CONCOMITANT	**Red yeast rice**	55	Female	Blood cholesterol increased, fatigue, **liver function test abnormal,** malaise, muscle spasms, myalgia, thyroid function test abnormal	Visited a Health Care Provider
6/11/2009	CONCOMITANT	Policosanol-**Red yeast rice**	55	Female	Abdominal pain, **hepatic enzyme increased,** hyperhydrosis, nausea, pancreatic disorder, pancreatitis	Hospitalization, Other Serious or Important Medical Event, Visited Emergency Room, Other Serious Outcome
